# Risk Factors for Treatment-Related Amenorrhea in Female Survivors of Childhood and Adolescent Cancer: 10-Year Experiences at Oncofertility Clinic in Korean Tertiary Center

**DOI:** 10.1089/jayao.2023.0132

**Published:** 2024-02-09

**Authors:** Soo Jin Park, Jung Yoon Choi, Hyoung Jin Kang, Yun Jeong Lee, Young Ah Lee, Ji Yeon Han, Sung Woo Kim, Hoon Kim, Seung-Yup Ku

**Affiliations:** ^1^ Department of Obstetrics and Gynecology, Seoul National University Hospital, Seoul, Republic of Korea.; ^2^ Department of Pediatrics, Seoul National University College of Medicine, Seoul National University Children's Hospital, Seoul, Republic of Korea.; ^3^ Seoul National University Cancer Research Institute, Seoul, Republic of Korea.; ^4^ Department of Pediatrics, Seoul National University Children's Hospital, Seoul, Republic of Korea.; ^5^ Departments of Obstetrics and Gynecology, Seoul National University College of Medicine, Seoul, Republic of Korea.; ^6^ Institute of Reproductive Medicine and Population, Medical Research Center, Seoul National University, Seoul, Republic of Korea.

**Keywords:** oncofertility, pediatric, fertility, survivorship

## Abstract

**Purpose:**

This study investigates the impact of gonadotoxic cancer treatment on treatment-related amenorrhea (TRA) and hormonal status in pediatric and adolescent females who underwent fertility preservation (FP) consultation.

**Methods:**

A retrospective review was conducted on 143 females under 21 with cancer referred to the FP clinic at Seoul National University Hospital between 2011 and 2022. We analyzed variables, including age, menarche status, cancer type, and treatment. Subsequently, subjects were evaluated to identify clinical factors affecting TRA at 1-year intervals following the completion of treatment. Upon cancer diagnosis, all patients received FP counseling and underwent semiannual evaluations for menstrual resumption and hormonal status.

**Results:**

The median age at diagnosis was 15; menarche was reported in 76.9%. Bone sarcoma (16.1%) and acute lymphoblastic leukemia (14.7%) were predominant. Most consultations (74.8%) occurred pretreatment. After FP consultations, 9.8% of patients underwent oocyte cryopreservation, and 99.3% used gonadotropin-releasing hormone agonists during systemic chemotherapy. One year after treatment completion, TRA was shown in 29.4% of this cohort. Cyclophosphamide-equivalent dose >4000 mg/m^2^ (adjusted odds ratio [aOR], 2.279; 95% confidence interval [CI]; 1.018–5.105, *p* = 0.045) and pelvic irradiation (aOR, 16.271; 95% CI, 1.545–171.408; *p* = 0.020) were independent clinical factors predicting TRA.

**Conclusion:**

The study delineates the clinical factors affecting TRA in pediatric and adolescent cancer survivors, revealing the significant impact of specific treatment. The data highlight the critical role of personalized oncofertility consultations in this demographic, offering valuable insights for designing targeted FP strategies at tertiary centers.

## Introduction

Cancer in pediatric and adolescent populations presents a unique challenge for health care systems worldwide, with increasing prevalence.^1,2^ Although advancements in diagnostic methods and treatment options have led to increased survival rates among these younger patients, the life-altering side effects of cancer therapies often extend to reproductive health and fertility.^3,4^ As survival rates improve, there is an increasing focus on enhancing the quality of life for survivors, an aspect of which is the ability to conceive and bear children.^5^

In this context, oncofertility, a field that straddles oncology and reproductive medicine, is prominent. It addresses the complex reproductive issues facing cancer patients, allowing them to maintain their fertility potential and make informed decisions about their future reproductive health.^6^ While there have been significant strides in adult oncofertility, the literature remains scant regarding the effects of pediatric and adolescent cancer treatments on fertility.^7,8^ Most pediatric cancer survivors are expected to reach adulthood, and many will have fertility as one of their chief long-term concerns.^1,9^

Several factors, including the type and stage of cancer, age at diagnosis, and the treatment modality used, can cause ovarian follicular loss, leading to diminished ovarian reserves and premature ovarian insufficiency.^10–12^ Therefore, pediatric and adolescent cancer survivors should be referred to fertility specialists and offered fertility preservation (FP) options. Various patient-specific factors, including age and pubertal development, influence FP techniques. In prepubertal patients, ovarian tissue cryopreservation emerges as the preferred modality, which obviates the need for hormonal induction.^13,14^ For patients who have reached pubertal maturation, additional methodologies such as oocyte or embryo cryopreservation can be employed.^13,14^ Concurrently, pharmacological interventions using gonadotropin-releasing hormone (GnRH) agonists or agonists are often utilized to mitigate chemotherapy-induced ovarian damage.^13,14^ While several studies have investigated FP techniques, such as oocyte or embryo cryopreservation in adult females, fewer studies have focused on pediatric and adolescent populations.^15^

Even fewer reports have specifically examined the amenorrhea rates in these younger populations and the consequent impact on fertility.^10^ Our study aims to elucidate the clinical significance of FP in adolescent and pediatric female cancer survivors by retrospectively reviewing the fertility outcomes of a cohort of females under 21, diagnosed and treated for various cancers and referred for FP at Seoul National University Hospital (SNUH).

## Method

### Ethics clearance

Ethics approval was obtained from the SNUH Institutional Review Board, ensuring adherence to ethical standards and patient privacy (approval no. 2307-126-140). Due to the study's retrospective nature and minimal participant risk, the requirement for written consent was waived.

### Patients and baseline data

This retrospective cohort study, conducted at SNUH between 2010 and 2022, focused on female patients diagnosed with malignancies (International Classification of Diseases [ICD]-10: C00–C99) before the age of 21. The focus was on individuals who underwent chemotherapy or radiotherapy as core components of their cancer treatments. Upon referral, all patients received tailored counseling on their FP options. The study targeted individuals with at least 12 months of post-treatment follow-up to ensure reliability in assessing treatment-related amenorrhea (TRA). Exclusions applied to patients who relapsed within this period were lost to follow-up or had incomplete medical records. Furthermore, only those consultations specifically related to post-treatment fertility were considered, while unrelated fertility cases were omitted. Among patients with gynecologic malignancies, those who had undergone fertility-sparing surgeries like ovarian cystectomy or unilateral salpingo-oophorectomy were included, while individuals who had nonfertility-sparing procedures such as bilateral salpingo-oophorectomy or total hysterectomy were excluded.

**FIG. 1.**
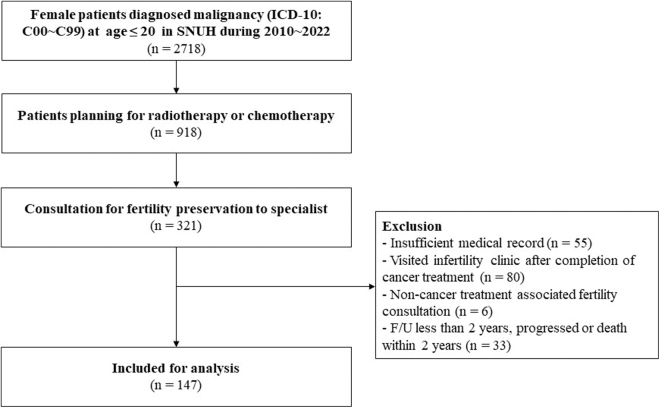
Study flowchart.

A total of 143 patients meeting eligibility criteria were included, as illustrated in Figure 1. Extracted data encompassed diverse demographics (age at diagnosis, last visit, menarche status, age at menarche, body mass index [BMI], cancer type, malignancy extent, and follow-up period), treatment specifics (chemotherapy, radiotherapy, hematopoietic stem cell transplantation, disease status, and timing of FP referrals), as well as FP details. Cyclophosphamide equivalent dose (CED) score was calculated to measure the potential gonadotoxic effects of various chemotherapy agents in cancer treatment. The calculation involves converting the doses of different agents into an equivalent dose of cyclophosphamide, known for its gonadotoxicity.^16^ The formula to calculate CED is as follows: CED (mg/m^2^) = Cyclophosphamide cumulative dose + (Ifosfamide cumulative dose × 0.244) + (Melphalan cumulative dose × 40) + (Busulfan cumulative dose × 8.823) + (Procarbazine cumulative dose × 0.857) + (lomustine [CCNU] cumulative dose × 16) + (carmustine [BCNU] cumulative dose × 15).

### FP consultation and post-treatment assessment

After the diagnosis of cancer, patients were referred to the FP clinic from the oncology part and were counseled on FP options such as oocyte cryopreservation, ovarian tissue cryopreservation, and GnRH agonist, depending on the urgency of treatment, age, status of puberty, and individual situations. For analysis, consultation timing was categorized into three distinct points: “before treatment,” “after surgery,” and “after initiation of chemotherapy.” The interval between cancer diagnosis and consultation was calculated in days. Collected data included trial of oocyte cryopreservation, usage of GnRH agonist, hormone levels, including anti-Müllerian hormone (AMH), follicle-stimulating hormone (FSH), estradiol (E_2_), and luteinizing hormone (LH), both before and after treatment, antral follicular count (AFC) assessed through pelvic ultrasonography, and resumption of menstruation. Detailed information on the stimulation protocol, total retrieved oocytes, cryopreserved mature oocytes, and the rate of oocyte maturity was recorded.

### Treatment-related amenorrhea

The study analyzed factors linked with cancer TRA after 1 year of treatment completion. Additionally, the impact of specific treatments (e.g., carboplatin, cisplatin, cyclophosphamide, ifosfamide, and busulfan) and radiation (pelvic and brain) on TRA likelihood was assessed using odds ratios (OR), adjusted OR, and 95% confidence intervals (CI).

### Data analysis

Data analysis utilized SPSS Version 26.0 (IBM Corp., NY). Descriptive statistics summarized demographics, treatment modalities, and FP patterns. Associations were evaluated using Chi-square or Fisher's exact tests for categorical variables, and Mann–Whitney *U* tests for continuous variables. Logistic regression assessed factors contributing to TRA. Statistical significance was set at *p* < 0.05.

## Result

In the patient demographics section (Table 1), the median age at cancer diagnosis was 15 years (range: 2–20), while the median age at the last visit was 20 years (range, 5–35). About 76.9% of patients had reached menarche at the time of cancer diagnosis, with a median age at menarche of 12 years (range, 9–18). The distribution of cancer types varied, with bone sarcoma (*n* = 23, 16.1%), acute lymphocytic leukemia (*n* = 21, 14.7%), and non-Hodgkin lymphoma (*n* = 20, 14.0%) being the most common. The extent of cancer status was categorized as localized in 21.7% of cases, regional in 27.3%, and distant in 17.5%. The median follow-up period was 50.4 months (range, 12.1–146.6). The most frequent treatment approach was chemotherapy (±surgery) at 74.1%, followed by combined chemotherapy and radiotherapy (±surgery) at 25.9%. Hematopoietic stem cell transplantation was undergone by 25.9% of patients. A significant percentage showed no evidence of disease after first-line treatment (85.3%), while others had recurrences (6.3%) or succumbed to disease progression (8.4%).

**Table 1.** Patient Demographics

Characteristics	Value
Age at cancer diagnosis (years), median (range)	15 (2–20)
Age at last visit (years), median (range)	20 (5–35)
Menarche at cancer diagnosis, *n* (%)	110 (76.9)
Age at menarche (years), median (range)	12 (9–18)
BMI (kg/m^2^), median (range)	20.5 (0.2–32.4)
Cancer type, *n* (%)	
Bone sarcoma	23 (16.1)
Acute lymphocytic leukemia	21 (14.7)
Non-Hodgkin lymphoma	20 (14.0)
Ovarian malignancy	17 (11.9)
Acute myelocytic leukemia	16 (11.2)
Hodgkin lymphoma	11 (7.7)
Brain tumor	11 (7.7)
Soft tissue sarcoma	9 (6.3)
Ewing sarcoma	5 (3.5)
Peripheral primitive neuroectodermal tumor	1 (0.7)
Etc.^a^	9 (6.3)
Extent of malignancy, *n* (%)	
Localized	31 (21.7)
Regional	39 (27.3)
Distant	25 (17.5)
Not available	48 (33.6)
Follow-up period (months), median (range)	50.4 (12.1–146.6)
Type of treatment, *n* (%)	
Chemotherapy only (± surgery)	106 (74.1)
Radiotherapy only (± surgery)	0
Chemotherapy and radiotherapy (± surgery)	37 (25.9)
Radiation dose	
Pelvic radiation (gy), median (range)	24 (12–66)
Total body irradiation (gy), median (range)	12 (12–12)
Brain radiation (gy), median (range)	60 (27–77.4)
Hematopoietic stem cell transplantation, *n* (%)	37 (25.9)
Disease status at last visit, *n* (%)	
No evidence of disease after first-line treatment	122 (85.3)
Recurred	9 (6.3)
Death due to progression	12 (8.4)

^a^ Includes tongue cancer (*n* = 1), Wilms Tumor (*n* = 1), breast cancer (*n* = 1), cervical cancer (*n* = 1), nasopharyngeal cancer (*n* = 1), neuroblastoma (*n* = 1), pancreatic cancer (*n* = 1), and gastric cancer (*n* = 1).

BMI, body mass index.

Moving on to FP referral and reproductive outcomes, depicted in Table 2, most consultations occurred before cancer treatment (*n* = 107, 74.8%), and the median interval from cancer diagnosis to consultation was 7 days. Only a small portion of cases (*n* = 14, 9.8%) underwent oocyte cryopreservation, and among GnRH agonists, leuprorelin was predominantly used (*n* = 106, 73.4%). Hormone levels and AFCs were evaluated both before and after treatment, and the Wilcoxon test showed a significant reduction of AMH levels (median, 3.1 vs. 0.1 ng/L; range, 0–16.2 vs. 0–11.1 ng/L; *p* < 0.001), increased FSH levels (median, 3.4 vs. 5.85 mIU/mL; range, 0–72.0 vs. 0.6–167 mIU/mL; *p* < 0.001), increased LH levels (median, 1.9 vs. 3.85 mIU/mL; range, 0.1–68.9 vs. 5.0–46.8 mIU/mL; *p* < 0.001), and a deceased AFC (median, 14 vs. 6; range, 0–87 vs. 0–51; *p* = 0.002), while no change in E_2_ levels. After 1 year of treatment completion, menstruation resumption was detected in 101 (70.6%) patients.

**Table 2.** Clinical and Hormonal Characteristics of Pediatric and Adolescent Females Who Underwent Fertility Preservation Counseling

Characteristics (*n* = 143)	Value
Timing for consultation, *n* (%)	
Before treatment	107 (74.8)
After surgery	22 (15.4)
After the initiation of chemotherapy	14 (9.8)
Interval from cancer diagnosis to consultation (days) median (range)	7 (0–435)
Oocyte cryopreservation, *n* (%)	
Oocyte retrieval before chemotherapy	7 (4.9)
Oocyte retrieval after chemotherapy	7 (4.9)
Not done	129 (90.2)
GnRH agonist, *n* (%)	
Leuprorelin	106 (73.4)
Goserelin	33 (23.1)
Decapeptyl	3 (2.1)
Not done	1 (0.7)
Hormone level before treatment, median (range)	
AMH (ng/L)	3.1 (0–16.2)
FSH (mIU/mL)	3.4 (0–72)
E2 (pg/ml)	29 (2.3–215)
LH (mIU/mL)	1.9 (0.1–68.9)
AFC before treatment (*n*), median (range)	10 (0–87)
Hormone level 1 year after treatment, median (range)	
AMH (ng/L)	1.1 (0–11.1)
FSH (mIU/mL)	5.85 (0.6–167)
E2 (pg/ml)	32.5 (1.1–250)
LH (mIU/mL)	3.85 (5–46.8)
AFC after treatment (*n*), median (range)	6 (0–51)
Interval from the last gonadotoxic treatment to menstruation (months), median (range)	6.86 (1.08–83.02)
Amenorrhea	
Amenorrhea at 1 year after treatment completion	42 (29.4)
Menstruation resumption 1 year after treatment completion	101 (70.6)

AFC, antral follicular count; AMH, anti-müllerian hormone; E_2_, estradiol; FSH, follicle-stimulating hormone; GnRH, gonadotropin-releasing hormone; LH, luteinizing hormone.

In the outcomes of controlled ovarian hyperstimulation (COH) cycles displayed in Supplementary Table S1, the random start COH was performed in 6 (42.9%) patients. The median number of total oocytes retrieved was 14 (range, 2–36), and the median number of total mature oocytes cryopreserved was 11 (range, 1–32).

Table 3 elucidates the clinical factors correlated with TRA in the study cohort. Notably, the analysis revealed no statistically significant differences in age and BMI distribution between the groups. However, a busulfan dose >450 mg/m^2^ was significantly more common in patients who did not experience menstrual resumption. While no significant differences were observed concerning specific treatment factors such as carboplatin, cisplatin, cyclophosphamide, ifosfamide, and other alkylating agents—including procarbazine, CCNU, and BCNU—pelvic irradiation (11.9% vs. 1.1%, *p* = 0.002) and hematopoietic stem cell transplantation (61.9% vs. 14.9%, *p* < 0.001) were significantly associated with TRA. Additionally, high CED scores (>4000 mg/m^2^) also demonstrated a significant correlation with increased rates of TRA 1 year after treatment (61.9% vs. 36.6%, *p* = 0.009).

**Table 3.** Clinical Factors by Fertility Impairment

	TRA (*n* = 42)	Resumption of menstruation (*n* = 101)	*p*
Age at diagnosis			0.094
≤15 years	30 (71.4)	57 (56.4)	
>15 years	12 (28.6)	44 (43.6)	
BMI			0.705
≤20 kg/m^2^	21 (50)	47 (46.5)	
>20 kg/m^2^	21 (50)	54 (53.5)	
Carboplatin			0.393
>2000 mg/m^2^	4 (9.5)	15 (14.9)	
≤2000 mg/m^2^	38 (90.5)	86 (85.1)	
Cisplatin			0.540
>500 mg/m^2^	1 (2.4%)	4 (4.0)	
≤500 mg/m^2^	41 (97.6%)	97 (96.0)	
Cyclophosphamide			0.257
>4000 mg/m^2^	11 (26.2)	18 (17.8)	
≤4000 mg/m^2^	31 (73.8)	83 (82.2)	
Ifosfamide			0.706
>17,000 mg/m^2^	6 (14.3)	17 (16.8)	
≤17,000 mg/m^2^	36 (85.7)	84 (83.2)	
Busulfan			<0.001
>450 mg/m^2^	10 (23.8)	0 (0)	
≤450 mg/m^2^	32 (76.2)	101 (100)	
Other alkylating agents^b^			0.107
Yes	0	6 (5.9)	
No	42 (100)	95 (94.1)	
CED^c^			0.009
>4000 mg/m^2^	26 (61.9)	37 (36.6)	
>2000 and ≤4000 mg/m^2^	2 (4.8)	20 (19.8)	
≤2000 mg/m^2^	14 (33.3)	44 (43.6)	
Brain irradiation	4 (9.5)	3 (3.0)	0.098
Pelvic irradiation	5 (11.9)	1 (1.0)	0.009
Total body irradiation	2 (4.8)	0 (0)	0.085
Hematopoietic stem cell transplantation	26 (61.9)	15 (14.9)	<0.001

^a^ Restoration group includes patients who do not exhibit high FSH, low E2, and resume menstruation or clinically expected to begin menarche.

^b^ Includes procarbazine, CCNU, and BCNU.

^c^ The CED score is calculated using the following equation: CED (mg/m^2^) = cyclophosphamide cumulative dose + (ifosfamide cumulative dose × 0.244) + (melphalan cumulative dose × 40) + (busulfan cumulative dose × 8.823) + (procarbazine cumulative dose × 0.857) + (CCNU cumulative dose × 16) + (BCNU cumulative dose × 15).

BCNU, carmustine; CCNU, lomustine; CED, cyclophosphamide equivalent dose; TRA, treatment-related amenorrhea.

Table 4 examines the OR for various clinical factors affecting the risk of TRA. Age, BMI >20 kg/m^2^, and pretreatment AMH levels were not significantly associated with TRA. The use of carboplatin and cisplatin at specific dosage thresholds also failed to demonstrate any significant impact, with *p*-values of 0.604 and 0.643, respectively. However, a CED >4000 mg/m^2^ was associated with a significantly increased risk, showing an adjusted OR of 2.279 (95% CI, 1.018–5.105; *p* = 0.045). Pelvic irradiation had an even more pronounced effect, with an adjusted OR of 16.271 (95% CI, 1.545–171.408; *p* = 0.020). Other factors, including carboplatin, cisplatin, cyclophosphamide, ifosfamide doses, and brain irradiation, did not show statistically significant associations with the risk of TRA.

**Table 4.** Risk of Treatment-Related Amenorrhea by Clinical Factors

	OR (95% CI)	*p*	Adjusted OR (95% CI)	*p*
Age	1.0 (0.9–1.1)	0.536	1.0 (0.9–1.1)	0.702
BMI >20 kg/m^2^	0.9 (0.4–1.8)	0.870	0.7 (0.3–1.5)	0.330
Pretreatment AMH	1.0 (0.9–1.1)	0.976	1.0 (0.9–1.1)	0.707
Carboplatin >2000 mg/m^2^	0.6 (0.2–2.0)	0.604	0.5 (0.1–1.7)	0.264
Cisplatin >500 mg/m^2^	0.6 (0.1–5.5)	0.643	0.6 (0.1–5.5)	0.612
CED >4000 mg/m^2^	2.8 (1.3–5.9)	0.006	2.3 (1.0–5.1)	0.045
Pelvic irradiation	14 (2–120)	0.019	16 (2–171)	0.020
Brain irradiation	3.4 (0.7–16.1)	0.117	4.4 (0.9–22.6)	0.074

CI, confidence interval; OR, odds ratio.

## Discussion

This study significantly contributes to the literature on FP in pediatric and adolescent female patients undergoing cancer treatment. Conducted at a single tertiary center associated with a children's hospital over 12 years, it provides detailed information about the impact of treatment modalities on TRA, especially in specific populations who underwent comprehensive FP counseling. Also, the diversity of cancer types echoed findings from earlier research, emphasizing the complexity of this patient group.^17–19^ These highlights contribute significantly to understanding FP in this unique patient population.

The most compelling findings in our study relate to the roles of high CED and pelvic irradiation in influencing TRA outcomes. High CED scores (>4000 mg/m^2^) and pelvic irradiation were significantly correlated with TRA, suggesting that they may be valuable markers for assessing the risk of fertility impairment in this population. These results are consistent with previous studies showing that CED affects female fertility at doses above 4000 or 8000 mg/m^2^ in most studies in a dose-dependent manner.^10,16^

Regarding radiation, the study's findings align with established knowledge, emphasizing the notable link between ovarian exposure to pelvic irradiation and a heightened risk of TRA. This reaffirms the well-documented gonadotoxic impact of pelvic radiation on ovarian tissue, underscoring the importance of cautious treatment planning to mitigate ovarian damage.^10,16,20^ While options like ovarian transposition have been considered in some contexts, it is essential to approach such interventions with caution, especially given the limited evidence available for their efficacy in younger demographics. More research is warranted to substantiate such recommendations for this particular age group.

Conversely, the lack of a significant relationship between brain irradiation and TRA observed in this study could be attributed to the limited number of patients subjected to brain irradiation, thereby hindering the ability to detect statistical significance. This finding diverges from prior research that has indicated a more evident connection between brain radiation and compromised fertility.^21^

In our study, age at diagnosis did not emerge as a significant factor affecting TRA in patients diagnosed with cancer before age 21. This is particularly noteworthy, as the relationship between age at cancer diagnosis and premature nonsurgical menopause is not well studied in this younger demographic.^10,16^ This observation, although intriguing, necessitates a nuanced interpretation. A considerable segment of our participants was yet to reach menarche during diagnosis, and given our follow-up constraints, it is unclear how many might naturally reach menarche subsequently. This potential variance might inadvertently distort the perceived correlation between age and TRA, underscoring the need for circumspect conclusions.

In the older population, for patients over 15 years of age at the time of cancer diagnosis, it has been reported that the risk of ovarian insufficiency increases with age.^22^ Additionally, the postchemotherapy level of AMH has been shown to decrease with advancing age at diagnosis in adult breast cancer.^23^ This suggests that the effect of age on ovarian function decreases due to cancer treatment and may differ in ovarian development status in the pediatric or adolescent population and adult cancer patients.

In our investigation, we utilized TRA at 1-year post-treatment as a surrogate marker for fertility impairment, a methodology that has been previously employed in numerous studies.^24,25^ In the realm of breast cancer research, it has been observed that 70%–80% of patients resume menstruation by the 1-year mark, with the majority either resuming their menstrual cycle by the end of the second year or not resuming it at all.^24,26^ However, in our study, we had a large population that was difficult to assess for TRA at year 2 because they were not followed up until 2 years or because they recurred within 2 years and started other treatments, so we evaluated TRA at 1 year as a surrogate marker for fertility after treatment, and this should be taken into account when interpreting our results. Consequently, the decision to use the 1-year post-treatment TRA as a fertility marker demands careful consideration during result interpretations.

One of the limitations tied to our study design was the exclusion of participants lost to follow-up or those with incomplete records. It is conceivable that individuals who experienced the resumption of their periods shortly after treatment might have been less inclined to revisit the clinic compared with those whose menstrual cycles did not resume or initiate within the year post-treatment. This exclusion might introduce a bias, and it is imperative to recognize that our findings could be influenced by such biases. We acknowledge the potential implications of this design decision and stress the importance of viewing our results in light of these considerations.

Furthermore, a year post-treatment, we observed a decline in AMH levels, coupled with elevations in FSH and LH. These hormonal changes with AFC measurements provide a robust framework for evaluating ovarian function following cancer treatment, in accordance with existing literature.^10,27,28^ The decrease in AMH and AFC as surrogate markers of ovarian hypofunction after gonadotoxic treatment correlates well with previous studies.^29,30^ The timing of E_2_ and FSH tests in this study may not accurately represent ovarian reserve either, given the lack of consistency in test timing relative to the menstrual cycle, which can be influenced by factors such as amenorrhea or varying intervals between treatment completion and assessment.^31^ These aspects accentuate the inherent complexities in post-treatment ovarian function evaluation, necessitating a balanced interpretation of our findings.

Concerning FP consultations, the majority occurred before cancer treatment initiation, aligning with the growing consensus on the importance of early discussions about FP options.^13,14^ However, the limited uptake of oocyte cryopreservation or no uptake of ovarian cryopreservation among patients indicated a persistent challenge in effectively informing patients about these possibilities. In this study, most patients referred to the fertility clinic agreed to use a GnRH agonist. However, only a limited number of patients underwent oocyte cryopreservation, and none underwent ovarian tissue cryopreservation.

In our cohort, patients were evenly divided between those who chose the random start protocol before gonadotoxic treatment and those who commenced COH post-treatment. This choice is often multifaceted, influenced by treatment urgency and overall health. For instance, the need for immediate treatment and medical conditions like poor general health or pancytopenia could complicate the decision, given the associated risks like hemorrhage, infection, anesthetic complications, and thrombotic events postoocyte retrieval.^14^

Although the literature suggests the utility of multiple consultations for informed FP decision making, the practicality of this within a tight treatment schedule is debatable.^32^ Nevertheless, post-treatment follow-ups at FP clinics provide a platform for patients to receive further consultation, particularly when their disease status has stabilized, thereby facilitating a more nuanced approach to FP decisions. Regarding oocyte cryopreservation outcomes, we observed comparable results to those in adult patients with a median age of 33 who underwent COH for FP.^33^ These findings suggest the viability of COH as a strategy for adolescents and young adults despite the unique challenges inherent to these populations.

As for ovarian tissue cryopreservation, once deemed experimental according to the 2018 American Society of Clinical Oncology (ASCO) guidelines,^13^ the most recent 2020 guidelines from the European Society of Human Reproduction and Embryology (ESHRE) now recognize it as an established FP option.^14,34,35^ This shift in guidelines might account for the absence of patients opting for ovarian tissue cryopreservation in our study period.

The inherent limitations of a retrospective study design, including the risks of selection bias and incomplete data, should be acknowledged as they may compromise the generalizability of our results. The relatively small sample size could limit the statistical power, potentially influencing the detection of significant associations. Additionally, the study primarily focuses on TRA at 1-year post-treatment as a surrogate marker for fertility without capturing other important fertility outcomes such as live birth rates or assisted reproductive technologies.

In conclusion, this study uniquely focuses on the FP considerations for young female cancer patients, a demographic often under-represented in the literature. Our key findings indicate that high CED of alkylating agents and pelvic irradiation significantly impact TRA. Interestingly, age at diagnosis was not a determining factor for TRA in this group, contrasting with what is often observed in older female cancer patients. Additionally, despite the limited evidence for their efficacy in young patients, GnRH agonists were commonly used in our study population. While this study has limitations, such as its retrospective design and small sample size, it offers significant clinical implications, suggesting the need for tailored approaches to FP consultations and treatments. This study opens avenues for improved care and quality of life for young female cancer survivors by aligning with and expanding upon existing literature.

## Supplementary Material

Supplemental data
