# Supplementary material for: Risk Factors for Treatment-Related Amenorrhea in Female Survivors of Childhood and Adolescent Cancer: 10-Year Experiences at Oncofertility Clinic in Korean Tertiary Center
Source: J Adolesc Young Adult Oncol. 2024 Feb 9;13(1):162–9. doi: 10.1089/jayao.2023.0132 (PMC10877397; doi:10.1089/jayao.2023.0132)
Supplement: Supplemental data [file Supp_TableS1.docx]

Supplementary Table 1. Outcomes of controlled ovarian hyperstimulation cycles

| **Variables (n = 16)** | **Value** |
| --- | --- |
| **Type of controlled ovarian hyperstimulation (n, %)** |  |
| Random start, GnRH antagonist protocol | 6 (42.9) |
| Random start, letrozole protocol | 1 (7.1) |
| Conventional | 8 (50) |
| **Number of total oocytes retrieved (n, median, range)** | 14 (2-36) |
| **Number of total mature oocytes cryopreserved (n, median, range)** | 11 (1-32) |
| **Mature oocyte rate (%, median, range)** | 83.3 (20-100) |

Abbreviations; GnRH, gonadotrophin-releasing hormone
